# Penta­cobalt(II) divanadium(III) tetrakis(diphosphate), Co_5_V_2_(P_2_O_7_)_4_

**DOI:** 10.1107/S1600536813008507

**Published:** 2013-04-10

**Authors:** Anna Bronova, Robert Glaum, Christian Litterscheid

**Affiliations:** a Institut für Anorganische Chemie, Universität Bonn, Gerhard-Domagk-Strasse 1, 53121 Bonn, Germany

## Abstract

Co_5_V_2_(P_2_O_7_)_4_ was crystallized by chemical vapour transport using HCl as transport agent. Its crystal structure is isotypic to that of Fe^II^_5_Fe^III^_2_(P_2_O_7_)_4_ and can be regarded as a member of the thortveitite structure family with corrugated layers of metal–oxygen polyhedra extending parallel to (010). Significant occupational disorder between cobalt(II) and vanadium(III) is observed. Four of the five cation sites are occupied by both cobalt and vanadium. The fifth cation site (Co1) is occupied by cobalt only. Sites Co1, *M*3 and *M*4 are located on twofold axes. Sites Co1, *M*2, *M*3 and *M*4 show o­cta­hedral coordination by oxygen; *M*5 has a square-pyramidal environment.

## Related literature

For related structures, see: Cruickshank *et al.* (1962 ▶); Gossner & Mussgnug (1929 ▶); Krishnamachari & Calvo (1972 ▶); Litterscheid (2009 ▶); Malaman *et al.* (1992 ▶); Palkina *et al.* (1985 ▶); Stefanidis & Nord (1984 ▶); Zachariasen (1930 ▶). For the preparation, see: Binnewies *et al.* (2012 ▶); Litterscheid (2009 ▶).

## Experimental

### 

#### Crystal data

Co_5_V_2_(P_2_O_7_)_4_
*M* *_r_* = 1092.29
Orthorhombic, 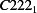

*a* = 8.3551 (4) Å
*b* = 9.7067 (5) Å
*c* = 23.8555 (11) Å
*V* = 1934.69 (16) Å^3^
*Z* = 4
Mo *K*α radiation
μ = 5.92 mm^−1^
*T* = 293 K
0.08 × 0.08 × 0.08 mm

#### Data collection

Stoe IPDS 2T diffractometer
Absorption correction: multi-scan (Blessing, 1995 ▶) *T*_min_ = 0.495, *T*_max_ = 0.647
9445 measured reflections
3800 independent reflections
2832 reflections with *I* > 2σ(*I*)
*R*_int_ = 0.082

#### Refinement

*R*[*F* ^2^ > 2σ(*F* ^2^)] = 0.046
*wR*(*F* ^2^) = 0.110
*S* = 0.92
3800 reflections
201 parameters
1 restraint
Δρ_max_ = 1.02 e Å^−3^
Δρ_min_ = −1.17 e Å^−3^
Absolute structure: Flack (1983 ▶), 2275 Friedel pairs
Flack parameter: −0.02 (2)

### 

Data collection: *X-AREA* (Stoe & Cie, 2002 ▶); cell refinement: *X-AREA*; data reduction: *X-RED* (Stoe & Cie, 2002 ▶); program(s) used to solve structure: *SHELXS97* (Sheldrick, 2008 ▶); program(s) used to refine structure: *SHELXL97* (Sheldrick, 2008 ▶); molecular graphics: *DIAMOND* (Brandenburg, 2008 ▶); software used to prepare material for publication: *WinGX* (Farrugia, 2012 ▶).

## Supplementary Material

Crystal structure: contains datablock(s) I, global. DOI: 10.1107/S1600536813008507/br2223sup1.cif

Structure factors: contains datablock(s) I. DOI: 10.1107/S1600536813008507/br2223Isup2.hkl

Additional supplementary materials:  crystallographic information; 3D view; checkCIF report

## Figures and Tables

**Fig. 1.**
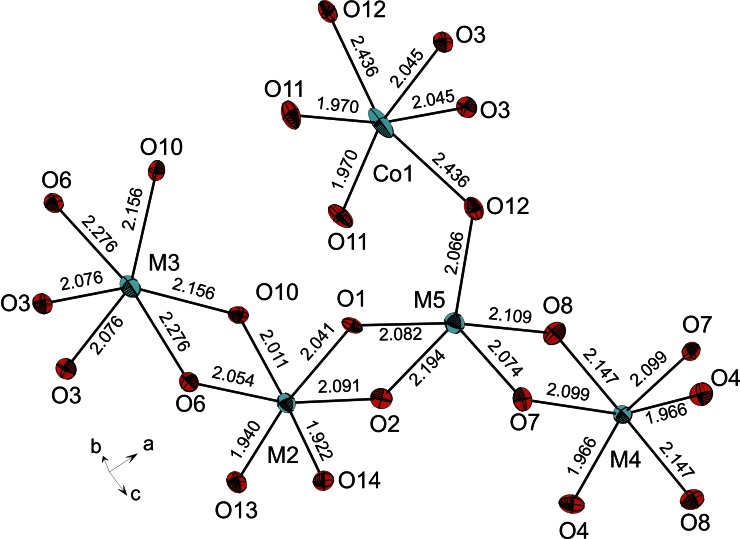
ORTEP style representation of metal-oxygen coordination polyhedra in Co5V2(P2O7)4. M1: Co, M2: Co0.45V0.55, M3: Co0.86V0.14, M4: Co0.69V0.31, M5: Co0.77V0.23. Ellipsoids at 50% probability level.

**Fig. 2.**
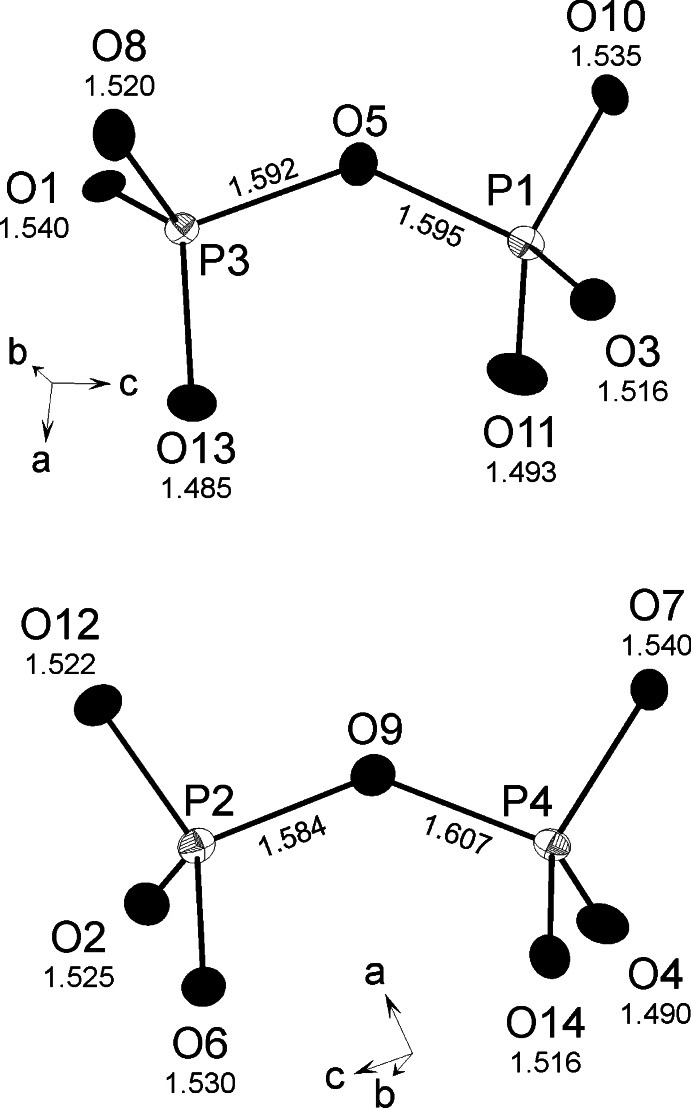
ORTEP style representation of the pyrophosphate groups. Ellipsoids at 50% probability level.

**Fig. 3.**
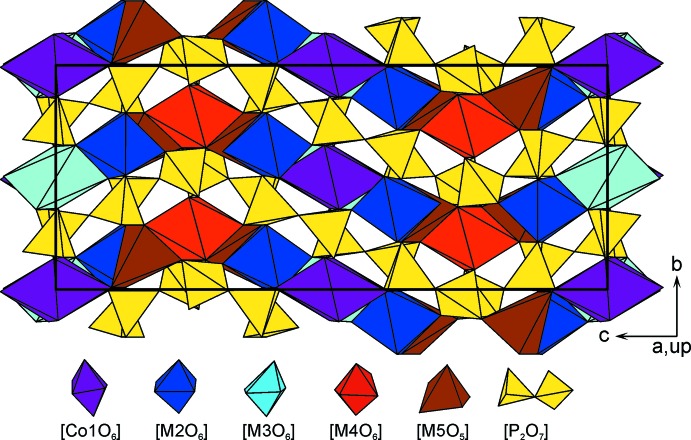
Projection along the a axis of the crystal structure of Co5V2(P2O7)4 with schematic coordination polyhedra. M1: Co, M2: Co0.45V0.55, M3: Co0.86V0.14, M4: Co0.69V0.31, M5: Co0.77V0.23.

**Fig. 4.**
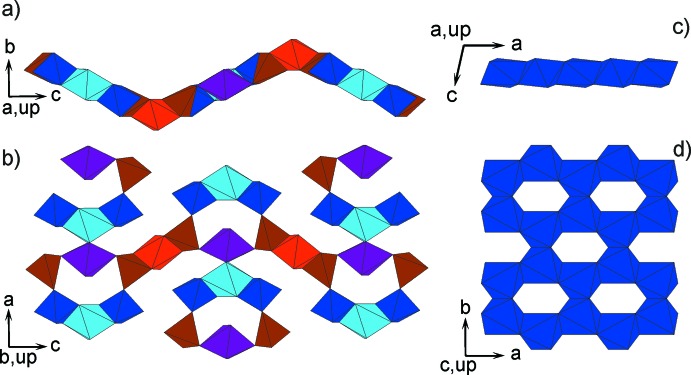
Comparison of the metal-oxygen co-ordination polyhedra and of their linkage in the crystal structures of Co5V2(P2O7)4 and Mn2P2O7. The same colour scheme as in Fig. 3 has been used.

## supplementary crystallographic information

### Comment

Equilibrium investigations in the ternary system CoO/V_2_O_3_/P_2_O_5_ revealed three new phosphates: Co^II^_3_V^III^_4_(PO_4_)_6_, Co^II^_3_V^III^_2_(P_2_O_7_)_3_, and Co^II^_5_V^III^_2_(P_2_O_7_)_4_ (Litterscheid, 2009).

Co^II^_5_V^III^_2_(P_2_O_7_)_4_ crystallizes in the orthorhombic space group *C*222_1_. The crystal structure is isotopic to that of Fe^II^_5_Fe^III^_2_(P_2_O_7_)_4_ (Malaman *et al.*, 1992). It consists of P_2_O_7_ groups and five independent polyhedra [*M*O_n_] (n = 5 or 6; Fig. 1). Four of the five cation sites are occupied by both cobalt and vanadium, two lying on a two-fold axis. The fifth cation site (Co1) is occupied by cobalt only. Sites Co1, M3 and M4 are located on two-fold axes. Sites Co1, M2, M3 and M4 show octahedral coordination by oxygen. M5 has a square-pyramidal enviroment. The metal oxygen polyhedra form corrugated layers (stacked along the *b*-axis). These layers are separated by P_2_O_7_ groups (Fig. 3).

The pyrophosphate groups display bridging angles (P1,O5,P3)=134.8 ° and (P2,O9,P4)=135.2 °. The displacement parameters for O5 and O9 do not exhibit any anomalies. The pyrophosphate group (P1P3O_7_) shows a staggered conformation while (P2P4O_7_) has an almost eclipsed one (Fig.2).

Allowing for disorder in the crystal structure of Co_5_V_2_(P_2_O_7_)_4_ led to significantly improved residuals. This refinement revealed disorder for four metal sites whereas the fifth site Co1 is fully occupied by cobalt (see table). The mean inter-atomic distances *d*(M-O) for the sites with mixed metal occupancy range from 2.01 Å to 2.17 Å. The average distance *d*(M-O) = 2.15 Å is found for [Co1O_6_]. All distances *d*(M-O) are in agreement with those already known from other cobalt(II) (Krishnamachari and Calvo, 1972) and vanadium(III) (Palkina *et al.*, 1985) phosphates.

The crystal structure of Co^II^_5_V^III^_2_(P_2_O_7_)_4_ shows close similarity to the thortveitite structure family (Gossner & Mussgnug, 1929; Cruickshank *et al.*, 1962; Zachariasen *et al.*, 1930). This relation is visualized in Fig. 4 by comparison of the structures of Co^II^_5_V^III^_2_(P_2_O_7_)_4_ and Mn_2_P_2_O_7_ (Stefanidis *et al.*, 1984). With respect to the composition of the latter pyrophosphate 5/8 of the Mn^2+^ sites are occupied by Co^2+^, 2/8 by V^3+^ and 1/8 remains empty for charge balance. In contrast to the honeycomb network of [MnO_6_] octahedra (Fig. 4) in Mn_2_P_2_O_7_ the polyhedra [*M*O_n_] in Co^II^_5_V^III^_2_(P_2_O_7_)_4_ show lower connectivity. This follows from the presence of octahedral voids and the reduced coordination number for polyhedron [*M*5O_5_].

### Experimental

For the synthesis of Co^II^_5_V^III^_2_(P_2_O_7_)_4_ a pellet was prepared of thoroughly ground V_4_(P_2_O_7_)_3_ (82.97 mg) and Co_2_P_2_O_7_ (166.99 mg). This pellet was transferred into a silica tube and sealed under vacuum. Heating at 1173 K for three days led to a single phase product. Pink single-crystals of Co_5_V_2_(P_2_O_7_)_4_ were obtained by chemical vapour transport (Binnewies *et al.*, 2012) in a temperature gradient 1273 → 1173 K for seven days using HCl as transport agent. HCl was obtained by *in situ* reaction of NH_4_Cl (2.7 mg) and PtCl_2_ (19.9 mg). In agreement with chemical vapour transport of other phosphates by HCl(g) as transport agent, we suggest the following transport reaction:

Co_5_V_2_(P_2_O_7_)_4_(*s*) + 16 HCl(*g*) = 5 CoCl_2_(*g*) + VCl_2_(*g*) + VCl_4_(*g*) + 2 P_4_O_10_(*g*) + 8 H_2_O(*g*)

### Refinement

Cobalt/vanadium disorder has been refined assuming full occupancy of metal sites *M*1 to *M*5. To maintain charge balance the refinement was constrained to a total of 20 Co^2+^ and 8 V^3+^ in the unit cell. The refinement indicated no disorder for site *M*1, for which in the final refinement cycles full occupancy by cobalt was assumed. Displacement parameters for sites with mixed occupancy Co/V were constrained to be identical for Co^2+^ and V^3+^. No hint on racemic twinning was observed.

Even after several hundred refinement cycles the occupancy ratio Co:V for site M2 showed a rather large value of 0.01 for the ratio shift/esd. Since there are no other hints on hidden problems with the crystal structure we think that this indicator just reflects the problems generally encountered when refining occupancy factors for atoms of similar atomic number. Actually, it was quite unexpected that the refinement of the mixed occupancies Co/V proceeded without further problems.

### Crystal data

**Table:**

Co_5_V_2_(P_2_O_7_)_4_	*F*(000) = 2100
*M**_r_* = 1092.29	*D*_x_ = 3.75 Mg m^−^^3^
Orthorhombic, *C*222_1_	Mo *K*α radiation, λ = 0.71073 Å
Hall symbol: C 2c 2	Cell parameters from 4330 reflections
*a* = 8.3551 (4) Å	θ = 1.0–29.1°
*b* = 9.7067 (5) Å	µ = 5.92 mm^−^^1^
*c* = 23.8555 (11) Å	*T* = 293 K
*V* = 1934.69 (16) Å^3^	Isometric, pink
*Z* = 4	0.08 × 0.08 × 0.08 mm

### Data collection

**Table:**

Stoe IPDS 2T 2-circle goniometer diffractometer	3800 independent reflections
Radiation source: sealed X-ray tube, 12 x 0.4 mm long-fine focus	2832 reflections with *I* > 2σ(*I*)
Graphite monochromator	*R*_int_ = 0.082
profile data from θ/2θ scans	θ_max_ = 34.1°, θ_min_ = 3.6°
Absorption correction: multi-scan (Blessing, 1995)	*h* = −12→12
*T*_min_ = 0.495, *T*_max_ = 0.647	*k* = −13→15
9445 measured reflections	*l* = −28→35

### Refinement

**Table:**

Refinement on *F*^2^	Secondary atom site location: difference Fourier map
Least-squares matrix: full	*w* = 1/[σ^2^(*F*_o_^2^) + (0.0575*P*)^2^] where *P* = (*F*_o_^2^ + 2*F*_c_^2^)/3
*R*[*F*^2^ > 2σ(*F*^2^)] = 0.046	(Δ/σ)_max_ = 0.058
*wR*(*F*^2^) = 0.110	Δρ_max_ = 1.02 e Å^−^^3^
*S* = 0.92	Δρ_min_ = −1.17 e Å^−^^3^
3800 reflections	Extinction correction: *SHELXL97* (Sheldrick, 2008)
201 parameters	Extinction coefficient: 0.00114 (15)
1 restraint	Absolute structure: Flack (1983), 2275 Friedel pairs
Primary atom site location: structure-invariant direct methods	Flack parameter: −0.02 (2)

### Special details

**Table:**

Experimental. To reach the desired data resolution with the given experimental setup the image plate had been tilted by 15 degrees. This is also the explanation for the asymmetrie in the upper and lower limits of observed *hkl* values.
Geometry. All e.s.d.'s (except the e.s.d. in the dihedral angle between two l.s. planes) are estimated using the full covariance matrix. The cell e.s.d.'s are taken into account individually in the estimation of e.s.d.'s in distances, angles and torsion angles; correlations between e.s.d.'s in cell parameters are only used when they are defined by crystal symmetry. An approximate (isotropic) treatment of cell e.s.d.'s is used for estimating e.s.d.'s involving l.s. planes.
Refinement. Refinement of *F*^2^ against ALL reflections. The weighted *R*-factor *wR* and goodness of fit *S* are based on *F*^2^, conventional *R*-factors *R* are based on *F*, with *F* set to zero for negative *F*^2^. The threshold expression of *F*^2^ > σ(*F*^2^) is used only for calculating *R*-factors(gt) *etc*. and is not relevant to the choice of reflections for refinement. *R*-factors based on *F*^2^ are statistically about twice as large as those based on *F*, and *R*- factors based on ALL data will be even larger.

### Fractional atomic coordinates and isotropic or equivalent isotropic displacement parameters (Å^2^)

**Table:**

	*x*	*y*	*z*	*U*_iso_*/*U*_eq_	Occ. (<1)
Co1	−0.08177 (13)	0	−0.5	0.0231 (3)	
Co2	−0.06997 (9)	−0.35558 (9)	−0.38065 (4)	0.01028 (16)	0.3452 (5)
V2	−0.06997 (9)	−0.35558 (9)	−0.38065 (4)	0.01028 (16)	0.6548 (5)
Co3	0.29241 (13)	0	−0.5	0.0162 (3)	0.934 (19)
V3	0.29241 (13)	0	−0.5	0.0162 (3)	0.066 (19)
Co4	0	−0.71981 (12)	−0.25	0.0106 (3)	0.72 (2)
V4	0	−0.71981 (12)	−0.25	0.0106 (3)	0.28 (2)
Co5	0.31161 (9)	−0.36011 (9)	−0.35848 (4)	0.0146 (2)	0.827 (12)
V5	0.31161 (9)	−0.36011 (9)	−0.35848 (4)	0.0146 (2)	0.173 (12)
P1	0.09658 (15)	−0.29386 (13)	−0.49912 (6)	0.0084 (2)	
P2	0.11867 (16)	−0.05623 (14)	−0.37555 (6)	0.0088 (3)	
P3	0.10814 (15)	−0.64697 (13)	−0.37996 (6)	0.0075 (2)	
P4	0.23708 (16)	−0.46167 (13)	−0.23058 (6)	0.0088 (2)	
O1	0.1176 (4)	−0.4895 (4)	−0.37362 (15)	0.0091 (7)	
O2	0.1166 (5)	−0.2135 (4)	−0.37347 (17)	0.0128 (7)	
O3	0.1028 (4)	−0.1378 (4)	−0.49593 (17)	0.0119 (7)	
O4	0.1778 (5)	−0.5885 (4)	−0.25902 (18)	0.0148 (8)	
O5	0.0296 (5)	−0.3310 (4)	−0.55996 (17)	0.0161 (8)	
O6	−0.2299 (4)	−0.5048 (4)	−0.40502 (16)	0.0119 (7)	
O7	−0.1713 (4)	−0.8684 (4)	−0.27171 (17)	0.0121 (7)	
O8	−0.0110 (5)	−0.7125 (4)	−0.33991 (17)	0.0144 (8)	
O9	0.1241 (4)	−0.0051 (4)	−0.31260 (17)	0.0138 (7)	
O10	−0.0396 (4)	−0.3561 (4)	−0.46426 (16)	0.0116 (7)	
O11	−0.2444 (5)	0.1430 (5)	−0.48668 (19)	0.0174 (8)	
O12	0.4680 (4)	−0.4907 (5)	−0.39934 (16)	0.0132 (7)	
O13	−0.2302 (5)	−0.2113 (4)	−0.38130 (18)	0.0131 (7)	
O14	0.1101 (5)	−0.3849 (4)	−0.19772 (17)	0.0132 (7)	

### Atomic displacement parameters (Å^2^)

**Table:**

	*U*^11^	*U*^22^	*U*^33^	*U*^12^	*U*^13^	*U*^23^
Co1	0.0073 (4)	0.0060 (4)	0.0559 (9)	0	0	−0.0023 (5)
Co2	0.0092 (3)	0.0085 (3)	0.0131 (4)	0.0004 (3)	−0.0003 (3)	0.0004 (3)
V2	0.0092 (3)	0.0085 (3)	0.0131 (4)	0.0004 (3)	−0.0003 (3)	0.0004 (3)
Co3	0.0109 (5)	0.0116 (5)	0.0260 (7)	0	0	−0.0022 (5)
V3	0.0109 (5)	0.0116 (5)	0.0260 (7)	0	0	−0.0022 (5)
Co4	0.0102 (5)	0.0075 (5)	0.0140 (6)	0	0.0005 (4)	0
V4	0.0102 (5)	0.0075 (5)	0.0140 (6)	0	0.0005 (4)	0
Co5	0.0118 (3)	0.0124 (4)	0.0197 (4)	−0.0005 (3)	0.0000 (3)	−0.0020 (3)
V5	0.0118 (3)	0.0124 (4)	0.0197 (4)	−0.0005 (3)	0.0000 (3)	−0.0020 (3)
P1	0.0081 (5)	0.0056 (5)	0.0114 (6)	0.0001 (4)	0.0015 (5)	0.0001 (5)
P2	0.0078 (5)	0.0064 (5)	0.0123 (7)	−0.0010 (4)	−0.0001 (5)	−0.0002 (5)
P3	0.0081 (5)	0.0043 (5)	0.0100 (5)	0.0012 (5)	−0.0004 (5)	0.0004 (5)
P4	0.0086 (6)	0.0070 (5)	0.0107 (6)	−0.0016 (4)	0.0009 (5)	0.0008 (4)
O1	0.0080 (14)	0.0060 (15)	0.0132 (18)	0.0023 (14)	0.0035 (14)	0.0015 (14)
O2	0.0141 (16)	0.0110 (17)	0.0133 (19)	−0.0008 (15)	0.0011 (16)	0.0030 (15)
O3	0.0112 (16)	0.0081 (15)	0.0162 (18)	0.0030 (14)	−0.0014 (16)	0.0017 (16)
O4	0.0163 (18)	0.0109 (17)	0.017 (2)	−0.0048 (15)	0.0022 (16)	−0.0004 (15)
O5	0.0145 (18)	0.019 (2)	0.0147 (19)	−0.0017 (15)	0.0007 (16)	−0.0063 (16)
O6	0.0106 (15)	0.0094 (16)	0.0157 (18)	−0.0007 (15)	0.0007 (14)	−0.0009 (14)
O7	0.0112 (15)	0.0103 (17)	0.0148 (18)	−0.0009 (14)	−0.0018 (14)	0.0043 (15)
O8	0.0179 (19)	0.0112 (18)	0.0142 (18)	−0.0027 (15)	−0.0001 (16)	−0.0004 (15)
O9	0.0102 (15)	0.0141 (17)	0.0171 (19)	−0.0014 (15)	0.0002 (14)	−0.0024 (16)
O10	0.0116 (16)	0.0128 (17)	0.0104 (17)	0.0014 (15)	0.0032 (14)	0.0001 (15)
O11	0.0107 (16)	0.0112 (17)	0.030 (2)	0.0046 (15)	0.0018 (16)	0.0011 (17)
O12	0.0080 (15)	0.0157 (18)	0.0158 (19)	−0.0022 (14)	0.0008 (14)	0.0036 (16)
O13	0.0114 (16)	0.0065 (16)	0.021 (2)	0.0020 (13)	−0.0019 (16)	−0.0014 (15)
O14	0.0122 (16)	0.0170 (19)	0.0102 (17)	0.0015 (15)	0.0014 (15)	−0.0011 (14)

### Geometric parameters (Å, º)

**Table:**

Co1—O11	1.968 (4)	Co5—O12	2.065 (4)
Co1—O11^i^	1.968 (4)	Co5—O7^iv^	2.076 (4)
Co1—O3^i^	2.044 (4)	Co5—O1	2.082 (4)
Co1—O3	2.044 (4)	Co5—O8^iv^	2.109 (4)
Co2—O14^ii^	1.921 (4)	Co5—O2	2.192 (4)
Co2—O13	1.937 (4)	P1—O11^v^	1.493 (4)
Co2—O10	2.010 (4)	P1—O3	1.518 (4)
Co2—O1	2.043 (3)	P1—O10	1.533 (4)
Co2—O6	2.055 (4)	P1—O5	1.597 (4)
Co2—O2	2.089 (4)	P2—O12^vi^	1.520 (4)
Co3—O3^i^	2.075 (4)	P2—O2	1.527 (4)
Co3—O3	2.075 (4)	P2—O6^iv^	1.531 (4)
Co3—O10^iii^	2.156 (4)	P2—O9	1.582 (4)
Co3—O10^iv^	2.156 (4)	P3—O13^v^	1.489 (4)
Co3—O6^iii^	2.274 (4)	P3—O8	1.519 (4)
Co3—O6^iv^	2.274 (4)	P3—O1	1.538 (4)
Co4—O4^ii^	1.969 (4)	P3—O5^vii^	1.591 (4)
Co4—O4	1.969 (4)	P4—O4	1.490 (4)
Co4—O7	2.097 (4)	P4—O14	1.515 (4)
Co4—O7^ii^	2.097 (4)	P4—O7^iv^	1.539 (4)
Co4—O8	2.148 (4)	P4—O9^viii^	1.608 (4)
Co4—O8^ii^	2.148 (4)		
			
O11—Co1—O11^i^	92.7 (3)	O11^v^—P1—O3	111.7 (2)
O11—Co1—O3^i^	93.86 (16)	O11^v^—P1—O10	113.0 (2)
O11^i^—Co1—O3^i^	167.12 (17)	O3—P1—O10	113.1 (2)
O11—Co1—O3	167.12 (17)	O11^v^—P1—O5	113.6 (2)
O11^i^—Co1—O3	93.86 (16)	O3—P1—O5	106.4 (2)
O3^i^—Co1—O3	82.0 (2)	O10—P1—O5	98.3 (2)
O14^ii^—Co2—O13	89.68 (18)	O12^vi^—P2—O2	114.9 (3)
O14^ii^—Co2—O10	170.85 (19)	O12^vi^—P2—O6^iv^	112.1 (2)
O13—Co2—O10	94.66 (17)	O2—P2—O6^iv^	110.5 (2)
O14^ii^—Co2—O1	87.68 (16)	O12^vi^—P2—O9	104.3 (2)
O13—Co2—O1	172.15 (17)	O2—P2—O9	106.4 (2)
O10—Co2—O1	89.03 (15)	O6^iv^—P2—O9	108.1 (2)
O14^ii^—Co2—O6	93.29 (17)	O13^v^—P3—O8	115.6 (2)
O13—Co2—O6	93.32 (16)	O13^v^—P3—O1	111.9 (2)
O10—Co2—O6	78.44 (16)	O8—P3—O1	112.8 (2)
O1—Co2—O6	94.22 (15)	O13^v^—P3—O5^vii^	107.4 (2)
O14^ii^—Co2—O2	98.53 (17)	O8—P3—O5^vii^	103.9 (2)
O13—Co2—O2	92.24 (16)	O1—P3—O5^vii^	104.1 (2)
O10—Co2—O2	89.36 (16)	O4—P4—O14	114.2 (2)
O1—Co2—O2	80.85 (15)	O4—P4—O7^iv^	111.2 (2)
O6—Co2—O2	166.96 (16)	O14—P4—O7^iv^	112.9 (2)
O3^i^—Co3—O3	80.5 (2)	O4—P4—O9^viii^	108.3 (2)
O3^i^—Co3—O10^iii^	153.63 (15)	O14—P4—O9^viii^	107.6 (2)
O3—Co3—O10^iii^	95.61 (15)	O7^iv^—P4—O9^viii^	101.8 (2)
O3^i^—Co3—O10^iv^	95.62 (15)	P3—O1—Co2	125.8 (2)
O3—Co3—O10^iv^	153.62 (15)	P3—O1—Co5	131.1 (2)
O10^iii^—Co3—O10^iv^	98.8 (2)	Co2—O1—Co5	103.14 (16)
O3^i^—Co3—O6^iii^	82.97 (15)	P2—O2—Co2	131.7 (3)
O3—Co3—O6^iii^	89.84 (15)	P2—O2—Co5	130.2 (2)
O10^iii^—Co3—O6^iii^	70.88 (14)	Co2—O2—Co5	98.01 (17)
O10^iv^—Co3—O6^iii^	115.74 (14)	P1—O3—Co1	128.7 (2)
O3^i^—Co3—O6^iv^	89.84 (15)	P1—O3—Co3	131.9 (2)
O3—Co3—O6^iv^	82.97 (15)	Co1—O3—Co3	98.73 (16)
O10^iii^—Co3—O6^iv^	115.73 (14)	P4—O4—Co4	137.3 (3)
O10^iv^—Co3—O6^iv^	70.88 (14)	P3^vii^—O5—P1	134.8 (3)
O6^iii^—Co3—O6^iv^	170.60 (19)	P2^ix^—O6—Co2	129.6 (2)
O4^ii^—Co4—O4	99.4 (3)	P2^ix^—O6—Co3^ix^	122.1 (2)
O4^ii^—Co4—O7	87.52 (17)	Co2—O6—Co3^ix^	102.37 (16)
O4—Co4—O7	158.93 (16)	P2^ix^—O6—V3^ix^	122.1 (2)
O4^ii^—Co4—O7^ii^	158.93 (16)	Co2—O6—V3^ix^	102.37 (16)
O4—Co4—O7^ii^	87.52 (17)	P4^ix^—O7—V5^ix^	128.6 (2)
O7—Co4—O7^ii^	93.1 (2)	P4^ix^—O7—Co5^ix^	128.6 (2)
O4^ii^—Co4—O8	93.18 (17)	P4^ix^—O7—Co4	125.9 (2)
O4—Co4—O8	84.36 (17)	V5^ix^—O7—Co4	105.46 (17)
O7—Co4—O8	75.35 (15)	Co5^ix^—O7—Co4	105.46 (17)
O7^ii^—Co4—O8	107.37 (16)	P3—O8—V5^ix^	127.8 (2)
O4^ii^—Co4—O8^ii^	84.36 (17)	P3—O8—Co5^ix^	127.8 (2)
O4—Co4—O8^ii^	93.18 (17)	P3—O8—Co4	127.9 (2)
O7—Co4—O8^ii^	107.37 (16)	V5^ix^—O8—Co4	102.55 (18)
O7^ii^—Co4—O8^ii^	75.35 (15)	Co5^ix^—O8—Co4	102.55 (18)
O8—Co4—O8^ii^	176.2 (2)	P2—O9—P4^x^	135.3 (3)
O12—Co5—O7^iv^	113.79 (16)	P1—O10—Co2	129.1 (2)
O12—Co5—O1	92.30 (15)	P1—O10—V3^ix^	121.6 (2)
O7^iv^—Co5—O1	101.71 (15)	Co2—O10—V3^ix^	108.17 (17)
O12—Co5—O8^iv^	94.11 (16)	P1—O10—Co3^ix^	121.6 (2)
O7^iv^—Co5—O8^iv^	76.62 (15)	Co2—O10—Co3^ix^	108.17 (17)
O1—Co5—O8^iv^	173.51 (15)	P1^vi^—O11—Co1	150.7 (3)
O12—Co5—O2	142.34 (16)	P2^v^—O12—Co5	127.2 (2)
O7^iv^—Co5—O2	103.80 (15)	P3^vi^—O13—Co2	158.5 (3)
O1—Co5—O2	77.59 (14)	P4—O14—V2^ii^	134.3 (3)
O8^iv^—Co5—O2	96.63 (16)	P4—O14—Co2^ii^	134.3 (3)

Symmetry codes: (i) *x*, −*y*, −*z*−1; (ii) −*x*, *y*, −*z*−1/2; (iii) *x*+1/2, −*y*−1/2, −*z*−1; (iv) *x*+1/2, *y*+1/2, *z*; (v) *x*+1/2, *y*−1/2, *z*; (vi) *x*−1/2, *y*+1/2, *z*; (vii) *x*, −*y*−1, −*z*−1; (viii) −*x*+1/2, *y*−1/2, −*z*−1/2; (ix) *x*−1/2, *y*−1/2, *z*; (x) −*x*+1/2, *y*+1/2, −*z*−1/2.
